# Vulnerability to anxiety and depression symptoms co-occurring among adult patients at family medicine clinics in Najran University Hospital, Saudi Arabia

**DOI:** 10.4102/sajpsychiatry.v32i0.2567

**Published:** 2026-01-14

**Authors:** Nasser Saeed Alqahtani

**Affiliations:** 1 Department of Family and Community Medicine, College of Medicine, Najran University, Najran, Saudi Arabia

**Keywords:** anxiety, depression, co-occurring, vulnerability, family medicine, primary care

## Abstract

**Background:**

Depression and anxiety are pervasive mental health concerns worldwide that pose significant challenges to individuals and societies.

**Aim:**

The aim of the study is to investigate the prevalence of co-occurring depression symptoms and anxiety among adult patients receiving care

**Setting:**

Primary clinics within the Family and Community Medicine Department, in Najran University Hospital, Saudi Arabia

**Methods:**

A cross-sectional study was conducted in February 2024, recruiting 300 adult patients across seven primary care clinics serving diverse populations. Data were collected using a comprehensive questionnaire that included socio-demographics, medical history, and screening for anxiety and depression symptoms. Multivariate regression analysis was employed.

**Results:**

Most participants were aged 21–30 years (33.0%), predominantly female (64.0%), and Saudi (68.7%). Chronic diseases affected 26.4%, and 31.6% had a past medical history. Depression symptoms were reported by 21.7%, while 14% had anxiety, and 10.7% experienced both. Multivariate regression analysis revealed that stress, psychiatric conditions, herbal medication use, and sleep problems were independent risk factors for co-occurring symptoms.

**Conclusion:**

Co-occurring anxiety and depression symptoms were prevalent in the study population.

**Contribution:**

The findings advocate for comprehensive mental health strategies that priorities the early detection of co-occurring depression and anxiety, by considering factors essential for improving patient outcomes.

## Introduction

Depression and anxiety are prevalent mental health disorders that significantly impact individuals and societies worldwide.^1,2,3^ According to the 2019 Global Burden of Disease Study, they are the leading causes of disability among mental health disorders, highlighting their substantial global health burden.^1,2^ Despite available treatments, the prevalence of these conditions has not decreased since 1990, indicating an ongoing mental health crisis that necessitates urgent attention.^4^ Depression is characterised by persistent sadness and a lack of interest in previously enjoyable activities, while anxiety manifests as excessive worry and physiological arousal.^5^ There is a strong correlation between anxiety and depression, particularly generalised anxiety disorder (GAD) and major depression, with many individuals experiencing both conditions simultaneously.^6^ The co-occurrence of these disorders is associated with greater symptom severity and functional impairment. The consequences of these disorders extend beyond individual suffering, leading to disabilities and, in severe cases, suicide.^7^ These far-reaching consequences compromise quality of life and impose substantial economic burdens on society.

Many individuals seek help in primary care settings, where a significant comorbidity exists – up to 56% of those with depression also have anxiety.^8^ In Saudi Arabia, a notable proportion of individuals visiting primary healthcare facilities exhibit signs of mental health disorders, with depression being particularly prevalent at 20%.^1,9^ Access to mental healthcare remains a global challenge; in the United States, around 25% of those with mental illness struggle to obtain necessary services, while less than 35% of affected individuals in developing countries receive adequate care.^10,11^ Alarmingly, up to 50% of individuals with common mental disorders remain undiagnosed in primary care.^12^ Enhanced screening and diagnostic methods are crucial to address this issue. Family medicine clinics are often the first point of contact for individuals seeking healthcare, making them vital in addressing depression and anxiety.^5^

The coexistence of anxiety and depression symptoms can significantly impact patients’ quality of life, treatment adherence, and overall health outcomes. While research has extensively explored these conditions in general populations and specific disease groups, there is a notable gap in understanding their co-occurrence among adult patients attending family medicine clinics in Saudi Arabia, particularly in the Najran region.^13^ Najran’s unique socio-cultural context, characterised by strong family ties, tribal identity, and cultural stigma surrounding mental illness, may influence mental health vulnerability and symptom expression. Local cultural perceptions may also affect help-seeking behaviours, symptom reporting, and treatment management.^13^ Given that the primary care setting is often the first point of contact, understanding the prevalence and co-occurrence of anxiety and depression is crucial for early identification, culturally sensitive screening, and effective intervention.

Existing research in Saudi Arabia has primarily focused on single conditions, specialised populations, or urban centres.^13,14^ While a recent study in Najran reported depression and anxiety prevalence rates among cancer patients, there is limited research on the co-occurrence of these conditions in family medicine clinics. This study aims to fill this gap by exploring co-occurring anxiety and depression in a primary care setting, considering the local cultural context and socio-cultural characteristics that may influence mental health vulnerability and presentation in Najran. Therefore, this study aims to assess adult patients’ vulnerability to these conditions at family medicine clinics in Najran University Hospital, quantifying the risk of co-occurring anxiety and depression and identifying influencing factors.

## Research methods and design

### Study design and setting

A cross-sectional study was conducted in February 2024 at the Family and Community Medicine Department of Najran University Hospital in Saudi Arabia to assess co-occurring depression symptoms and anxiety among adult patients. This design facilitated the simultaneous collection of data on depression symptoms and anxiety screening, socio-demographic characteristics, and medical history within a specific timeframe across seven primary care clinics serving diverse populations.

### Sample size

A sample size of 300 participants was determined after consultation with a statistician, considering factors such as desired accuracy, expected prevalence of co-occurring disorders, and an acceptable margin of error.

### Participant selection

A simple approach was employed to select participants; all adult patients who visited any family medicine clinic during the study period were invited to participate. Participants who provided informed consent were consecutively included until the desired sample size was achieved to ensure representation of the target population.

### Study instrument

A comprehensive questionnaire was developed based on a thorough literature review and consultations with mental health experts. The questionnaire included questions on socio-demographic characteristics, medical history, and depression symptoms and anxiety screening. The first part of the questionnaire captured socio-demographic information, including age, sex, marital status, nationality, education, residency, family type, number of family members, employment status, workload, social relationships, economic status, and presence of stress. The second part focused on medical characteristics, including chief complaints, complaint duration, current illness progression, presence of chronic diseases, past medical history, psychiatric diseases, regular medication use, herbal treatment or supplement use, allergies, sleep problems, pregnancy and lactation status, smoking habits, nutrition, physical activity, and body weight. The questionnaire’s third part combined depression symptoms and anxiety screening using standardised tools. The Patient Health Questionnaire-2 (PHQ-2) served as a brief depression symptoms-screening tool, consisting of two questions focused on depressed mood and loss of interest over the past 2 weeks. The Generalised Anxiety Disorder 7-item scale (GAD-7) assessed anxiety through seven items covering various anxiety experiences during the same period.

This two-stage method efficiently identifies individuals needing further evaluation and potential interventions for depression symptoms. Previous studies with over 500 participants demonstrated the PHQ-2’s effectiveness in identifying major depressive disorder (MDD) and any depressive disorder with prevalence rates of 7% and 18%.^15^ The GAD-7 accurately identifies probable anxiety disorder cases and gauges anxiety severity, with higher scores correlating with impaired daily functioning and increased missed workdays because of disability. Despite the co-occurrence of anxiety and depression symptoms, factor analysis confirmed the GAD-7’s ability to differentiate between these conditions.^16^ While primarily designed for GAD, the GAD-7 has also demonstrated utility for assessing other anxiety disorders, including panic disorder, post-traumatic stress disorder, and social anxiety disorder.^17^

A PHQ-2 score of 3 or higher indicates an increased likelihood of depressive symptoms and warrants further evaluation using the PHQ-9, other diagnostic tools, or a structured clinical interview to confirm the diagnosis accurately.^15^ In the context of anxiety disorder screening, a threshold of 8 or higher on the scale is deemed appropriate for identifying potential cases, mandating subsequent diagnostic scrutiny to ascertain both the presence and specific subtype of anxiety disorder.

### Scoring criteria

A score of three or higher on the PHQ-2 was considered indicative of potential MDD, warranting further evaluation. For the GAD-7, a score of 8 or higher was considered appropriate for identifying potential cases of anxiety disorders.

### Questionnaire validation

The questionnaire was rigorously evaluated by three mental health experts to assess its validity and reliability. Based on their feedback, appropriate modifications were made to enhance the questionnaire’s validity and reliability and ensure its suitability for the study’s objectives.

### Data collection procedure

After thorough training, nurses incorporated the questionnaire into their initial patient intake processes. Patients who provided informed consent completed the questionnaire during a brief interview conducted by nurses, ensuring standardised data collection procedures.

### Statistical analysis

Data analysis was conducted using the Statistical Packages for Social Sciences (SPSS) version 26 to ensure robust statistical analyses to comprehensively address the study objectives. Descriptive statistics were used to summarise the categorical variables and presented as frequencies and percentages. The relationship between co-occurring anxiety and depression with socio-demographic and medical characteristics was analysed using the Chi-square test. Significant results were further analysed using a multivariate regression model to determine the independent risk factors; corresponding odds ratios and 95% confidence intervals (CIs) were calculated. The significance level was set to *p* < 0.05.

### Ethical considerations

Ethical clearance to conduct this study was obtained from the Faculty of Medicine at Najran University (No. CSR/NU/2023/1046). Ethical clearance and informed consent were obtained before the start of the survey.

## Results

The study found that most participants were aged 21–30 years (33.0%), predominantly female (64.0%), and married (52.1%). The majority were Saudi (68.7%) with university education, and 71.3% were unemployed, as shown in Table 1.

**TABLE 1:** Socio-demographic characteristics of participants (*N* = 300).

Variables	*n*	%
**Age group (years)**		
≤ 20	43	14.3
21–30	99	33.0
31–40	40	13.3
41–50	59	19.7
51–60	39	13.0
> 60	20	06.7
**Sex**		
Male	108	36.0
Female	192	64.0
**Marital status†**		
Single	120	41.7
Married	150	52.1
Divorced	08	02.8
Widowed	10	03.5
**Nationality**		
Saudi	206	68.7
Non-Saudi	94	31.3
**Educational level**		
Illiterate	27	09.0
Secondary or below	88	29.3
University education	154	51.3
Postgraduate	31	10.3
**Residence location**		
Rural	22	07.3
Semi-urban	29	09.7
Urban	249	83.0
**Family type†**		
Nuclear family	102	41.5
Extended family	144	58.5
**Number of family members**		
< 3	28	09.7
3–7	169	58.3
8–14	84	29.0
≥ 15	09	03.1
**Number of children**		
No	153	51.0
1–2	74	24.7
3–5	51	17.0
> 5	22	07.3
**Employment status**		
Unemployed	214	71.3
Government employee	53	17.7
Private employee	27	09.0
Non-profit	01	0.30
Self-employed	05	01.7

† , Respondents who did not provide their socio-demographic information were excluded from the analysis.

Regarding the medical characteristics of participants, chronic diseases were reported by 26.4% of participants, and 31.6% had a documented past medical history. Psychiatric illnesses affected 7.4% of patients. Regular medication usage was seen in 47.4%. Allergies were found in 13.8% of cases, and 35.4% reported sleep problems. Most were non-smokers (90.5%), while 73.3% consumed a mixed diet (Includes both plant-based foods and animal products, such as meats, dairy, and eggs, alongside a variety of fruits, vegetables, and grains). Physical activity levels ranged from none to mild (46.4%). Body weight distribution showed 39.7% normal weight, 33.6% overweight or obese, and 5.1% underweight.

**FIGURE 1:**
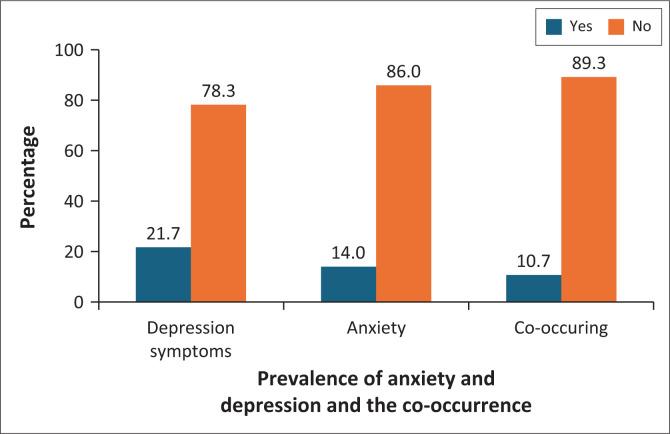
Prevalence of anxiety and depression and the co-occurrence of anxiety and depression.

Figure 1 shows that 21.7% of participants had depression symptoms, whereas 14% had anxiety. A significant proportion (10.7%) of patients experienced both conditions simultaneously. This highlights the prevalence of anxiety and depression in the population, with a substantial portion of patients exhibiting comorbid symptoms.

Table 2 illustrates the relationship between co-occurring anxiety and depression and the socio-demographic characteristics of participants. Individuals aged 30 years and younger exhibited higher prevalence rates compared to those over 30. Marital status, employment status, workload, and social relationships showed significant associations. Stress significantly correlated with a higher prevalence of co-occurring anxiety and depression among those reporting stress.

**TABLE 2:** Associations between co-occurring anxiety and depression and the socio-demographic characteristics (*N* = 300).

Factor	Co-occurring anxiety and depression				*P*-value‡
	Yes (*N* = 32)		No (*N* = 268)		
	*n*	%	*n*	%	
**Age group (years)**	-	-	-	-	< 0.001*
≤ 30	25	78.1	117	43.7	-
> 30	07	21.9	151	56.3	-
**Sex**	-	-	-	-	0.554
Male	10	31.3	98	36.6	-
Female	22	68.8	170	63.4	-
**Marital status†**	-	-	-	-	< 0.001*
Unmarried	26	83.9	112	43.6	-
Married	05	16.1	145	56.4	-
**Nationality**	-	-	-	-	0.222
Saudi	25	78.1	181	67.5	-
Non-Saudi	07	21.9	87	32.5	-
**Educational level**	-	-	-	-	0.918
Secondary or below	12	37.5	103	38.4	-
University or higher	20	62.5	165	61.6	-
**Family type†**	-	-	-	-	0.512
Nuclear family	10	35.7	92	42.2	
Extended family	18	64.3	126	57.8	
**Number of family members**	-	-	-	-	0.666
≤ 7	20	64.5	177	68.3	-
> 7	11	35.5	82	31.7	-
**Number of children**	-	-	-	-	0.169
Yes	12	37.5	135	50.4	-
No	20	62.5	133	49.6	-
**Employment status**	-	-	-	-	0.084
Unemployed	27	84.4	187	69.8	-
Employed	05	15.6	81	30.2	-
**Workload†**	-	-	-	-	0.285
Low	20	76.9	135	66.5	-
Moderate to intolerable	06	23.1	68	33.5	-
**Social relationships†**	-	-	-	-	< 0.001*
Healthy	19	61.3	222	87.1	-
Toxic	12	38.7	33	12.9	-
**Having stress**	-	-	-	-	< 0.001*
Yes	31	96.9	145	54.1	-
No	1	3.1	123	45.9	-

† , Respondents who did not provide their socio-demographic information were excluded from the analysis.

‡ , *p*-value has been calculated using the Chi-square test.

* , Significant at *p* < 0.05 level.

Table 3 examines the associations between co-occurring anxiety and depression and medical characteristics. Current illness progression, psychiatric co-morbidities, herbal treatments, and sleep problems were linked to increased co-occurring. Other factors showed no significant associations.

**TABLE 3:** Associations between co-occurring anxiety and depression and medical characteristics (*N* = 300).

Factor	Co-occurring anxiety and depression				*P*-value‡
	Yes (*N* = 32)		No (*N* = 268)		
	*n*	%	*n*	%	
**Duration of complaint (months)**	-	-	-	-	0.482
< 1	20	95.2	166	86.9	-
≥ 1	01	04.8	25	13.1	-
**Current illness progression†**	-	-	-	-	0.058
Improved	06	20.0	69	30.5	-
Same	16	53.3	131	58.0	-
Deteriorated	08	26.7	26	11.5	-
**Having chronic disease†**	-	-	-	-	0.142
No	27	84.4	182	72.2	-
Yes	05	15.6	70	27.8	-
**Past medical history†**	-	-	-	-	0.830
No	20	66.7	153	68.6	-
Yes	10	33.3	70	31.4	-
**Having a psychiatric disease†**	-	-	-	-	< 0.001*
No	19	63.3	243	96.0	-
Yes	11	36.7	10	04.0	-
**Using regular medications†**	-	-	-	-	0.953
No	17	53.1	133	52.6	-
Yes	15	46.9	120	47.4	-
**Using herbal treatment†**	-	-	-	-	0.001*
No	23	71.9	236	90.8	-
Yes	09	28.1	24	09.2	-
**Using supplement†**	-	-	-	-	0.314
No	25	78.1	221	85.0	-
Yes	07	21.9	39	15.0	-
**Having allergy†**	-	-	-	-	0.053
No	24	75.0	225	87.5	-
Yes	08	25.0	32	12.5	-
**Sleep problems†**	-	-	-	-	< 0.001*
No	06	18.8	182	70.3	-
Yes	26	81.3	77	29.7	-
**Smoking†**	-	-	-	-	0.057
Non-smoker	26	81.3	242	91.7	-
Smoker or Ex-smoker	06	18.8	22	08.3	-
**Nutrition†**	-	-	-	-	0.297
Healthy food	05	15.6	49	18.6	-
Unhealthy food	05	15.6	20	07.6	-
Mixed	22	68.8	195	73.9	-
**Physical activity†**	-	-	-	-	0.908
None	07	21.9	64	24.3	-
Mild	16	50.0	121	46.0	-
Moderate to vigorous	09	28.1	78	29.7	-
**Body weight†**	-	-	-	-	0.093
Normal	12	50.0	104	50.7	-
Overweight or obese	08	33.3	90	43.9	-
Underweight	04	16.7	11	05.4	-

† , Respondents who did not provide their socio-demographic information were excluded from the analysis.

‡ , *p*-value has been calculated using the Chi-square test.

* , Significant at *p* < 0.05 level.

Table 4 presents multivariate regression analysis identifying stress as a significant predictor of co-occurring anxiety and depression, with stressed participants nearly nine times more likely to experience co-occurring. Having a psychiatric disorder was also significantly associated, with affected individuals being over 15 times more likely to exhibit co-occurring. In addition, those using herbal treatments and experiencing sleep problems were four times more likely to show these symptoms. However, age, marital status, and social relationships showed no significant associations.

**TABLE 4:** Multivariate regression analysis to determine the significant independent risk factor for co-occurring anxiety and depression (*N* = 300).

Factor	AOR	95% CI	*p*-value
**Age group (years)**			
≤ 30	Ref	-	-
> 30	0.453	0.096–2.136	0.317
**Marital status†**			
Unmarried	Ref	-	-
Married	0.240	0.046–1.255	0.091
**Social relationships†**			
Healthy	Ref	-	-
Toxic	1.007	0.312–3.246	0.991
**Having stress**			
No	Ref	-	-
Yes	8.945	1.103–72.563	0.040*
**Having a psychiatric disease†**			
No	Ref	-	-
Yes	15.505	3.798–63.00	< 0.001*
**Using herbal treatment†**			
No	Ref	-	-
Yes	3.675	1.069–12.631	0.039*
**Sleep problems†**			
No	Ref	-	-
Yes	3.803	1.260–11.480	0.018*

AOR, adjusted odds ratio; CI, confidence interval.

† , Respondents who did not provide their socio-demographic information were excluded from the analysis.

* , Significant at *p* < 0.05 level.

## Discussion

This study examined the susceptibility of adult patients at family medicine clinics in Najran University Hospital to co-occurring anxiety and depression. The findings revealed a co-occurrence rate of 10.7%, with depression prevalence (21.7%) higher than that of anxiety (14%). These results provide valuable insights into the existing literature, especially in Saudi Arabia, where research on the co-occurrence of these mental health conditions has been limited. Given the high comorbidity and often indistinguishable symptoms of these disorders,^18^ there is a pressing need for enhanced screening and intervention strategies in primary care settings to effectively address these prevalent issues.

When comparing the study’s findings with those of previous studies, it is noteworthy that the rates of co-occurring varied across different regions and populations. For instance, a study conducted in Brazil reported a higher incidence of co-occurring, with anxiety prevalent among 74% of depressed patients and depression common among 61% of anxiety patients.^19^ Similarly, 27% of elderly individuals in the Netherlands had depression and coexisting general anxiety disorders.^20^ These variations highlight the complex interplay between anxiety and depression and underscore the need for tailored interventions that consider cultural, demographic, and contextual factors. Interestingly, this study identified several risk factors associated with coexisting anxiety and depression, including stress, psychiatric diagnoses, use of traditional medicine, and sleep disorders. These findings align with existing literature regarding the multifactorial nature of these conditions. However, the study did not find significant differences in co-occurring symptoms based on chronic conditions, medical history, or physical activity, which contrasts with De La Rosa et al. (2024) and underscores the need for further research across diverse populations and settings.^21^

Patients with mental health conditions are often at increased risk for comorbidities and frequently utilise healthcare services. Up to 80% of people worldwide use herbal medicine, with around 22% of patients in Australia seeking treatment for mental illness opting for herbal remedies.^22^ This highlights the importance of healthcare providers engaging in discussions about these practices with their patients. This study found that younger age, unmarried status, and toxic relationships predict co-occurring anxiety and depression, supporting previous research on the role of socio-demographic factors. However, other variables did not significantly impact these conditions, which is consistent with Beekman et al..^23^ Unlike other study found no significant link between age and depression, highlighting the need for further research on these complex relationships.^24^ Prina et al. found that comorbid conditions were primarily linked to higher disability scores, independent of sex and socioeconomic status.^25^

Furthermore, this study aligns with Hsu et al., identifying stress, psychiatric diagnoses, and sleep disorders as key risk factors for co-occurring anxiety and depression.^26^ This reinforces the understanding that these factors play a critical role in the development of both conditions, emphasising the need for targeted interventions that address these underlying issues. Notably, factors such as illness progression, medication intake, and dietary habits were not significant predictors, contrasting with existing literature and highlighting the need for a re-evaluation of these relationships in future research.

### Strengths and limitations

The study contributes valuable insights into anxiety and depression in Saudi Arabia, utilising validated tools and a representative sample. Limitations include self-reported data, a cross-sectional design, and a focus on one hospital, which may introduce bias. Another limitation is that positive PHQ-2 screening results were not followed by a structured clinical interview or PHQ-9 assessment to confirm depressive disorder. Therefore, the findings reflect a depression rather than clinically diagnosed depression, and the prevalence may be overestimated. Future research should include longitudinal studies.

## Conclusion

This study highlights the significant prevalence of co-occurring anxiety and depression among adult patients at Najran University Hospital, with 10.7% affected. Key risk factors include stress, psychiatric co-morbidities, herbal medicine use, and sleep problems, emphasising the need for integrated treatment approaches to address these interconnected issues. Further prospective studies are necessary to clarify the causal relationships among these variables. Comparisons with existing literature reveal variability in prevalence rates, underscoring the complexity of these disorders. Overall, the findings advocate for comprehensive mental health strategies that prioritise early detection while considering socio-demographic, medical, and cultural factors in assessment and management, which are crucial for effectively addressing these complexities and improving patient outcomes.
